# Tackling Cardiovascular Care Deserts in Romania: Expanding Population Access in Underserved Areas

**DOI:** 10.3390/healthcare12242577

**Published:** 2024-12-21

**Authors:** Alexandra Cioclu, Liliana Dumitrache, Alina Mareci, Mariana Nae

**Affiliations:** Faculty of Geography, University of Bucharest, 010041 Bucharest, Romania; alexandra.cioclu@s.unibuc.ro (A.C.); mariana.nae@geo.unibuc.ro (M.N.)

**Keywords:** cardiovascular care deserts, access, travel time, distance, aged population, Romania

## Abstract

Background: Cardiovascular deserts are areas that lack medical facilities, specialists and equipment to effectively diagnose, treat and manage cardiovascular diseases (CVDs). Romania registers the highest incidence and the highest mortality due to CVDs in Europe. Population ageing is a significant concern, as it increases the risk of CVDs and the demand for specialised care. Although almost 50% of Romanians still live in rural areas, most medical resources are concentrated in a few large cities, leaving large parts of the country underserved. Methods: This study used the Application Programming Interface (API) Matrix service from Google Maps and open data sources to identify cardiovascular (CV) deserts. Results: This research indicates that over 64% of the Romanian population resides in areas lacking CV care, having to travel more than 60 km and over 30 min to reach the nearest facility that offers specialised treatment. Moreover, 14% live in areas affected by a high degree of cardiovascular desertification. These areas are primarily located in northeastern, southern and western Romania. They experience higher mortality rates from CVDs and an ageing population, along with a shortage of general physicians and a scarcity of cardiologists. Conclusions: The identified cardiovascular deserts in this study overlap mountainous regions, the Danube Delta and remote rural areas with poor transportation infrastructure. Implementing telemedicine or mobile healthcare services, involving community healthcare workers and policy support could be solutions to expand access to specialised care in cardiovascular deserts.

## 1. Introduction

The term medical desert is becoming prevalent in recent discourse on the topic of healthcare-underserved areas [1,2]. The ubiquitous term associated with these areas is rural or the idea of rurality [3,4,5,6]. Isolation or remoteness is also used in these descriptions [7]. Essentially, while there is no widespread definition, medical deserts are areas where healthcare providers or services are limited or altogether unavailable [8]. The richness of studies on underserved areas in terms of healthcare is undeniable, but the idea of medical deserts is relatively new. This term has gained traction within academia and health policy actors due to its urgency, as it creates the idea of desolation in the reader’s mind [9]. Medical deserts are among the health workforce priorities for the European Union (EU) [10], with the 2020 agenda aiming to keep people healthy and active for longer [11] by reducing inequalities in health and obtaining inclusive economic growth [12]. Medical desertification is seen as a consequence of insufficient human resources or medical facilities, long waiting times, which generate delayed first contact with a doctor, disproportionate high costs for transport or other socio-cultural barriers [13]. Furthermore, remote or rural areas create difficult working conditions, which the health workforce tends to avoid [14], thus aggravating the medical desertification of those areas.

The process of defining or identifying medical deserts, mainly powered by the availability of data or sometimes existing regulations, differs across studies. Nonetheless, there are numerous commonalities across the board. In research on specific types of deserts, the defining factor is the lack of that specialised service, for example, maternity care providers in Louisiana [15], vascular surgeons [16] or surgeons in general [17] in two other US studies. Another important aspect is the relationship between the population and healthcare practitioners. Such examples can be cited when discussing cancer deserts [18] or gynaecologist deserts [19] in the US.

General practitioners (GPs) are considered gatekeepers of the healthcare system; often, their insight into patients’ medical history can successfully detect the first signs of health risks, including cardiovascular (CV) ones. Their availability and numbers are always part of defining medical deserts. Indicators such as the density of GPs per inhabitant were used in studies in France [20,21], Romania [9] and Poland [22].

The number of pharmacies in their role as suppliers of immediate tools for prevention and care, or respectively, population per pharmacy, have been considered in standalone studies on pharmacy deserts in the US [23] and as part of the defining characteristics of medical deserts in Romanian research [2]. Medication is essential in preventing and mitigating chronic diseases and their associated complications, and a scarcity of pharmacies brings about reduced medication adherence and increases the risk of CVD complications [24]. The importance of pharmacies, especially their absence, is strengthened by studies introducing the concept of “medication deserts” [25].

Hospitals represent one of the most cited healthcare units in studies that analyse medical deserts. The presence of hospitals, cumulated with the presence of GP offices/primary care units and pharmacies, was considered by Rosik et al. (2020) in the case of Poland [22], where a 30 min travel time threshold to reach a hospital unit was set. Nguyen et al. (2021) used the same 30 min travel time limit for defining hospital deserts in the US [26], with longer travel times seen as a burden on the patients. Savignat (2013) also considered that travel times longer than 30 min to a specialised care unit, such as a hospital, should define medical deserts [27].

In other studies on medical deserts, an index of all or some of these elements has been constructed, or values below the national average have been set as delineating boundaries [2]. Bonal et al. (2024) considered more than one factor in defining medical deserts; as such, they took into account the availability of GPs and primary care providers, travel time to specific units and population characteristics [21]. In the same way, Dubas-Jakóbczyk et al. (2024) explored the problem of medical deserts in Spain and considered the maximum distance of 50 km to a health facility, the area’s characteristics, health workers and the size of the population in defining them [14].

A recurring element that many studies (focused either on underserved areas or medical deserts) settle on is distance. Some authors discuss distance as an important finding [28,29,30,31,32,33]. Distance in these cases fluctuates depending on the study area and type of service measured, from 15 miles for primary care units [29] to more than 21 miles for cancer units in the US [31]. In a study specifically on pharmacy deserts, Wisseh et al. (2021) considered distance as a defining factor for the county of Los Angeles [34]. Similarly, a Pennsylvania-level study considered pharmacy deserts as areas where 3% of enrolees live more than 1 mile from one such unit [35], but Nguyen et al. (2021) [26] set a 10-mile limit, while another US national-level research study on pharmacy deserts combined distance with the presence of economically vulnerable population [36]. A study on trauma deserts in the case of Chicago set a 5-mile distance as an exclusion factor and created categories based on travel time [37].

Studies have mostly used travel time in hospital-centred analysis [38,39]. Vergier et al. (2017) identified medical deserts in France based on travel time, using slots from 10 min to more than 45 min to reach specific healthcare units. The authors emphasised these areas as an accumulation of difficulties [20]. Chevillard et al. (2018) considered travel time a defining characteristic, while other indicators, such as the number of consultations per inhabitant, play an essential role in hierarchising medical deserts [40].

Longer travel times to hospitals or emergency care services generate higher mortality levels in life-threatening situations, including CV ones [41,42]. Aneja et al. (2011) demonstrated that distances to cardiologists can reduce the probability of seeking consultations at symptom onset, lower adherence to follow-up visits and more complications [43]. A similar conclusion was reached by Uribe-Leitz et al. (2018) when analysing the causes of delayed diagnoses, which then necessitate complex surgeries [44]. Nicholl et al. (2007) proved that the average distance to emergency care services is increasing, naming causes such as the centralisation of medical services, financial and staff reasons or the need to use the available medical equipment effectively [45]. As car-to-hospital travel time estimated by GIS networks was proven to be similar to actual travel times, it is presently easier and more reliable to analyse travel times and distances using instruments such as API [19,27,32,46,47].

The absence of a clear definition of medical deserts leads to confusion and complicates efforts to address healthcare disparities. Without a uniform framework, researchers struggle to pinpoint specific barriers, such as insufficient healthcare facilities, long waiting times and socio-cultural obstacles that contribute to medical deserts, which in turn hampers effective resource allocation and policy formulation.

Access to healthcare services is considered an essential right in the EU [48], so identifying and analysing underserved areas is not a new endeavour. Delayed access to healthcare increases morbidity and mortality connected to acute and chronic illnesses such as cancer [49] or CVDs [50]. An issue when talking about healthcare-underserved areas is that applying the concept of accessibility means translating it into operational indicators, which can be difficult [51,52]. Clark et al. (2012), for example, constructed a remoteness index in an attempt to quantify potential accessibility to different types of healthcare units and different ranks of hospitals [53]. Both this study and Finster et al. (2023) recognised geography and healthcare unit distribution as essential mechanisms in this process [18]. The difference in distribution is clearly in favour of urban areas, as proven by multiple studies, with some important ones including Suditu et al. (2013) [54], and becomes more important when studying CVDs [55,56]. Thus, potential accessibility does not entirely translate into healthcare utilisation, yet it is a topic of many studies as it is relatively more straightforward to quantify. Potential accessibility is created by the spatial arrangement of an area’s healthcare and transport networks, which in turn depend on the uneven distribution of the geographical features of that terrain. In rural Kentucky, poor road conditions and higher elevations were linked to longer travel times to healthcare facilities, proving that geographical features directly impact access [57]. At the same time, research in Nepal indicated significant disparities in access to public health facilities, particularly in mountainous areas [58]. Eastern Europe has not been a major target of studies focusing on medical deserts. A very important contribution was made by Rosik et al. (2021) [22]. However, the lack of such studies is noted, for example, by Bes et al. (2024) [59] who do not mention any Eastern European countries in a systematic review of medical deserts. Previous geographical studies in Romania have explored the uneven distribution of healthcare facilities [60] or healthcare workforce [61], while other studies have focused on healthcare accessibility [62,63,64]. Two recent studies discussed medical deserts in Romania [2,13], highlighting significant gaps in understanding the concept. While studies on medical deserts provide valuable insights, their focus is rarely on Eastern Europe or Romania. Additionally, the identified Romanian studies do not tackle medical deserts related to CVDs.

CVDs are the leading cause of death in Romania; the mortality rate due to CVDs is one of the highest in EU countries [65]. A MediNET sentinel network project (2002) aiming at computerising GP practices in Romania identified that every fourth adult patient is a CV patient [66]. Ischemic heart disease is the leading cause of death through CVDs at 19.1%, double that of the EU, followed by stroke at 16.3% [65]. As CVDs have a complex typology, correctly identifying, monitoring or treating them demands highly specialised care. The 2010 National Invasive Program for Acute Myocardial Infarction Treatment developed in Romania aims to improve treatment for myocardial infarction. Currently, it functions only in nine hospitals located in Bucharest and some of the countries’ larger cities (Iași, Timișoara, Târgu Mureș and Cluj-Napoca) [67,68]. Thus, while high-quality treatment for acute myocardial infarction exists, it tends to be located in university hospitals, leading to the expansion of underserved areas.

CVDs generate a considerable financial burden due to treatment costs [69]. Therefore, cardiovascular desert research must revolve around the territorial distribution of hospitals and their ability to successfully treat CVDs and identify underserved areas from this perspective. Addressing this gap would not only shed light on the geographic disparities in CV care but is essential for developing comprehensive strategies that can effectively mitigate the impact of CVDs and ensure equitable access to healthcare services across Romania. As such, this study significantly contributes to the limited existing body of research by spanning a wide range of data from demographics to health infrastructure and supports efforts to improve health outcomes nationally.

This research is the first seeking to tackle CV deserts nationally. The objectives of this study are to identify and delineate CV deserts at the national level using a standard methodology and to hierarchise these areas considering the population’s vulnerability to CVDs and their needs for care, as well as the existing healthcare infrastructure.

## 2. Materials and Methods

### 2.1. Study Area

Romania is one of the countries with the largest population in the EU, with 19 million inhabitants in 2024 and a surface of 238,400 km^2^. Romania’s administrative structure consists of 3181 Local Administrative Units (LAU2), comprised of communes, municipalities and cities. The population of an LAU2 varies between 116 and 2,160,169 inhabitants [70]. Romania has an overdeveloped urban network, consisting of 320 cities, including the capital, Bucharest, with 2.1 million inhabitants, four cities with around 300,000 inhabitants and 225 smaller towns with fewer than 20,000 inhabitants [71]. This creates a significant imbalance between urban centres in Romania, particularly the capital and the next four to five cities, which house most of the healthcare services, including infrastructure, workforce and specialised care. The political and economic changes after the 1990s led to large cities undergoing modernisation and economic growth while medium and small towns underwent shrinking, facing depopulation, demographic ageing and poor infrastructure [72]. In small towns, the population’s access to essential services such as education and healthcare is limited due to the lack of specialised workforce, equipment and poor road infrastructure.

The geographic characteristics of the country include 27% mountainous regions, with altitudes ranging from 800 to 2500 m. Additionally, 42% of the land consists of hills and plateaus, while plains account for 30%. Wetlands comprise 5% of the total land surface [73]. In areas with poorly developed road infrastructure, the characteristics of these landforms often hinder access to healthcare services. The quality and density of Romania’s road system are inferior to those of other EU countries [74]. Specifically, it consists of 997 km (5.6%) of highways, 70 km (0.4%) of express roads, 35,046 km (40.6%) of county roads and 33,665 km (39.0%) of municipal roads. Only half are modernised, 26.7% are paved roads and 23.9% are paved for light traffic [75]. The Bucharest-Ilfov area stands out as the most developed in the country and benefits from advanced transport infrastructure and a robust service sector, which has attracted both domestic and foreign investments [76]. In contrast, the northeastern and southwestern parts face severe economic challenges, being among the regions with the highest shares of the population living at risk of poverty in the EU, struggling with high unemployment and out-migration [54,77,78].

Romania is experiencing a significant demographic decline and ageing population trends that have persisted for over three decades [79]. This decline is primarily attributed to a combination of low birth rates, high mortality rates and out-migration. Its demographic structure is shifting, with 19.7% of the population being over 65 years [80]. This increases the pressure on social and healthcare services. Although these demographic particularities demand high investments in healthcare provision, Romania spends a lower percentage of its GDP (6.3%) on healthcare than other EU countries [81].

Romania’s healthcare system functions primarily as a public, tax-funded model. Although, after 1990, efforts were made to reform it, the healthcare system consistently faces issues of low performance, insufficient funding, shortages of medical personnel and ineffective service delivery. Its hospital-care centricity contributes to inefficiencies within the healthcare system, as it fails to utilise GP offices and outpatient services adequately. The hospital network includes 546 hospital units, out of which 366 are public and 180 are private [82]. Despite the relatively high number of hospital units and their uniform geographic distribution across the country, not all hospitals can provide complex CV care. Population health outcomes reflect these geographic disparities in the distribution of CV healthcare facilities. Order no. 323/2011 [83] divides hospitals into five categories based on their level of competence and medical services complexity. Hospitals belonging to ranks I and II can offer complex care, while the III–V ranked hospitals provide basic medical care and often lack the resources and specialised workforce needed to manage complex diseases. These inequalities result from several attempts to reform the Romanian healthcare system. These policies are not unique to our country and mirror the policies elsewhere in southeast Europe [84].

### 2.2. Data

#### 2.2.1. Population Data

We considered 3181 LAU2 as points for assessing distance and travel time to the nearest CV hospital able to provide complex care. Data on the total population at the lowest administrative level and those over 65 years old were retrieved from the National Institute of Statistics (NIS) database and are publicly available [70]. The surface of each LAU2 was obtained from the National Agency for Cadastre and Real Estate Advertising (ANCPI) and is publicly available [85]. Data on the number of deaths due to CVDs were recovered from the NIS.

#### 2.2.2. Healthcare Data

From the total number of hospitals, we selected the 161 units that have a cardiology department. Out of these, 43 are included in the National Cardiovascular Disease Program, which means that they can offer complex CV care. These 43 hospitals generally belong to rank I–II. They are unevenly distributed across the country, many of which are located in Bucharest, the capital, and a few large cities such as Cluj-Napoca, Constanța, Iași and Timișoara. The competencies of hospitals in providing complex CV care and surgical capabilities were the criteria for inclusion in this study. General data related to the healthcare infrastructure in Romania are available in both the NIS and the Romanian Ministry of Health databases. The Romanian Ministry of Health provides data about hospital classification and their coordinates.

Data on GP offices in Romania are found in the NIS database and are publicly available [70]. Additionally, the number and addresses of GP offices at the LAU2 level were retrieved from the 41 County Health Insurance Houses (CHIHs). The number of GPs was collected from the NIS database, while the geographic locations of each practising GP were corroborated from the websites of the 41 CHIHs. A total of 203 websites were reviewed for data extraction. Since GPs are reimbursed for the treatments they provide if they have a framework contract with the National Health Insurance House, only the GPs active under that framework contract were included in this study.

Data on pharmacies at the national level were taken from the NIS database. Furthermore, the number and geographical locations of pharmacies at the LAU2 level were collected from the websites of the 41 CHIHs. This study only considered pharmacies contracted with the National Health Insurance House because this contract enables them to provide compensated or free medications based on prescriptions from GPs or specialists.

Data related to the cardiologist workforce can be found in the NIS database [70]. However, this database considers all medical staff, including administrative or scientific research personnel, often leading to overestimating the practising workforce. There is no comprehensive database that details the dimensions and distribution of the healthcare workforce across various healthcare facilities. Therefore, the number of practising cardiologists presented in this study at each CV hospital was collected directly from the hospital website.

### 2.3. Methods

This study identified areas referred to as ‘cardiovascular deserts’ based on the distance and travel time to a CV hospital that can provide complex care. Therefore, all LAU2 located more than 60 km away, where the population has to travel more than 30 min to access such specialised CV care, were designated as cardiovascular deserts. The 30 min travel time was chosen due to it being a recurring threshold in hospital-centred desert studies [22,26,27]. This observation is also strengthened by a European Climate and Health Observatory study that summarised the average travel time to the nearest hospital in Romania to be around 30 min [86]. Distance is often present in studies on underserved areas in terms of healthcare, or more specifically medical deserts, but its threshold varies depending on the type of medical desert presented [29,30,36,37,38,39]. While distance categories differ across studies, a common denominator is that an increased distance results in lower health outcomes. Studies in Europe and North America often consider travel times of 30–60 min to essential healthcare facilities, which translates to approximately 40–70 km depending on road and transport conditions [20,26,27,28,40]. The Romanian Government Emergency Ordinance No. 195/2002 states that ambulances do not have to respect speed limits, and all traffic participants must prioritise emergency vehicles as quickly as possible [87]. Therefore, in the context of Romania, where underserved areas experience limited healthcare access and transportation challenges, a 60 km distance represents a practical threshold reflecting the maximum reasonable travel distance that can be covered in 30 min. The 60 km and 30 min metrics were selected based on a combination of geographic accessibility standards and CVD emergency-specific considerations, and the two selections work as double validation conditions. A hierarchy of the identified cardiovascular deserts was created based on the population’s vulnerability to CVDs and their need for care, as well as the existing basic healthcare infrastructure.

#### 2.3.1. Identification and Geocodation of Cardiovascular Hospitals

The process of identifying the CV hospitals included in this study was based on their classification, meaning only hospitals that can provide complex CV care and are included in the Cardiovascular Disease National Program were considered. Geocoding each cardiovascular hospital involved using the coordinates provided by the Google Maps platform, which were validated with the Romanian Ministry of Health database and the hospitals’ websites. This third validation approach ensured accuracy and minimised errors, as multiple sources were cross-referenced to confirm the geocoded locations. For example, if a discrepancy arose between the Google Maps information and the one from the Ministry of Health database, further verification was performed using the hospitals’ websites. ArcGIS Pro 2.5.0 software was used to geocode the CV hospitals.

#### 2.3.2. Identification and Delineation of Cardiovascular Deserts

The Google Maps API, a service that provides the travel distance and time from an origin point (O) to a destination (D) (O–D travel time matrix), was used to identify the cardiovascular deserts. We assessed the most effective travel route suggested by Google for each LAU2 centroid based on its proximity to the nearest CV hospital that can provide complex care. Due to the recognised impact of traffic conditions on travel times and because Google Maps calculates travel time based on real-time traffic data, we considered the average travel time. Travel time and distance were calculated considering the car as the mode of transport. A total of 2223 LAU2 out of 3181 LAU2 have been identified in a radius of more than 30 min travel time and over 60 km distance from a selected CV hospital. These areas were categorised as ‘cardiovascular deserts’. All 2223 LAU2s were manually validated.

#### 2.3.3. Classification of Cardiovascular Deserts

To ensure a clear image of the areas dealing with low levels of health infrastructure and health personnel, we quantified the level of CV desertification.

The hierarchy of the cardiovascular deserts was established considering the population’s vulnerability to CVDs and their potential need for specialised care alongside the current basic healthcare infrastructure in the area. This process involved the construction of indexes based on multiple indicators. A common challenge in developing these indexes is the lack of a standard approach for integrating diverse data types measured with different metrics, making it difficult to assign relative importance to each indicator [88].

*The Population Vulnerability Index* (*IV_n_*) indirectly reflects the population’s needs for CV care. The *IV_n_* uses available data and is based on three indicators: CVD mortality rate, the percentage of the population over 65 years and population density. The CVD mortality rate serves as a direct indicator of disease burden and reflects the effectiveness of healthcare systems in managing CV chronic conditions. The percentage of the population over 65 years is particularly relevant due to their higher susceptibility to CVDs. Population density is an essential indicator in assessing the need for targeted intervention because it provides insights into population distribution in a specific area. A statistical approach to indicator choice involves determining the correlation between indicators and then including the less correlated ones to minimise redundancy [89]. Thus, we calculated a correlation matrix representing the Pearson correlation coefficients (Table 1).

The values of the three indicators included in the *IV_n_* were normalised using Min–Max Normalisation to a standard range between 0 and 1, and the sum of the absolute values of the correlations was used to attribute weight to each indicator, as shown in the formula:

$$w_{i}=\frac{\sum_{j=1,j\neq i\;}^{n}\left|rij\right|}{\sum_{k=1\;\;}^{n}\sum_{j=1,j\neq k\;}^{n}\left|rkj\right|}$$

$w_{i}$ = weight assigned to variable *i* in the Population Vulnerability Index.
*i*, *j*, *k* = variables.
$\left|rij\right|,\;\left|rkj\right|$ = absolute value of the Pearson correlation coefficient.

The resulting weight is ≈0.3526 for the percentage of the population over 65 years, ≈0.3728 for mortality rate due to CVDs and ≈0.2746 for population density. The *IV_n_* was calculated based on the formula:

$${IV}_{n}=\sum_{i=1}^{n}w_{i\;}\cdot \;X_{i\;}$$

*IV_n_* = Population Vulnerability Index.
*n* = total number of variables included.
$w_{i}$ = weigh assigned to variable *i*.
$X_{i}$ = value of variable *i* in the dataset.

The values of the *IV_n_* range from 0–1.48, with the lowest values (close to 0) representing reduced vulnerability to CVDs and low potential need for care, while higher values suggest higher vulnerability to CVDs and high need for CV care.

*The Basic Healthcare Infrastructure Index* (*IB_hi_*) highlights the different levels of scarcity of basic healthcare infrastructures for the identified CV deserts. It is based on four indicators. We calculated provider-to-population ratios for the number of identified GPs, GP offices and pharmacies and assigned scores based on the existence of a rank III, IV or V hospital in the area. Rank III–V hospitals were included in this index because even if they can only provide basic care, their presence can mitigate CV complications to a certain degree. Still, a potential patient needs to be transferred to a higher-rank CV hospital for more complex investigations and treatment, as the approach of a previous study confirms [63]. Scores differed depending on hospital rank. For example, a hospital with a rank of III received a score of 3, leading to a higher value of the *IB_hi_* because it has broader competency. Lower values of the *IB_hi_* correspond to a more precarious supply of healthcare infrastructure; as such, a rank IV hospital received a score of 2 and a rank V hospital received a score of 1. The normalised values were summed, and the *IB_hi_* was derived using the following formula:

$${IB}_{hi\;}=\sum_{i=1}^{n}X_{i\;}$$

${IB}_{hi}$ = Basic Healthcare Infrastructure Index.
$X_{i}$ = normalised values of the ratios.
*n* = total number of ratios.

The *IB_hi_* values range between 0 and 5.1, with low values illustrating a high degree of scarcity, lack or very low number of healthcare providers, while high values suggest more healthcare providers or the presence of a better-ranked hospital.

The *Cardiovascular Desertification Index* (*ID_cv_*) was constructed to hierarchise the cardiovascular deserts based on the population’s vulnerability to CVDs and need for care, as well as the identified basic healthcare infrastructure. Consequently, it was constructed using *IV_n_* and *IB_hi_* using the formula

$${ID}_{cv}=\frac{\sum_{i=1}^{n}w_{i\;\;}\cdot \;X_{i\;\;}}{(\sum_{i=1}^{n}w_{i\;\;}\cdot \;X_{i\;\;}+\sum_{i=1}^{n}\;X_{i\;\;})}$$

${ID}_{cv}$ = Cardiovascular Desertification Index.
$w_{i}$ = weigh assigned to variable *i*.
$X_{i}$ = value of variable *i* in the dataset.

The degree of desertification is illustrated by values ranging from 0 to 1. The highest values emphasise a scarcity of healthcare infrastructure and services, overlapping with high CVD vulnerability and need for care. At the other end of the spectrum, low *ID_cv_* values describe a slightly better situation, corresponding to more healthcare units combined with lower CVD vulnerability and potential needs for care. This hierarchy enhances comparability among the different cardiovascular desert areas identified.

## 3. Results

### 3.1. Uneven Geographic Distribution of Healthcare Infrastructure and Workforce in Romania

At the national level, the geographic distribution of healthcare units highlights significant disparities, particularly when comparing urban to rural areas. Conversely, rural areas are severely underserved, incorporating 40% of the GP offices and 20% of the pharmacies (Table 2). In 2023, 65,000 healthcare units were registered, with a striking 53,000 located in urban settings. Although the number of hospitals is significant, they follow an urban-centric distribution, with 90.9% of all hospitals, underscoring the concentration of healthcare resources in large cities [61].

Romania’s healthcare workforce is currently facing a significant shortage of medical personnel, which poses serious challenges. In 2023, there were over 72,000 physicians in the country, resulting in a ratio of 258 inhabitants per physician, below the EU average. Also, the country has 12,471 GPs practising under a framework contract with the National Health Insurance House, which makes them eligible for reimbursements. Moreover, 85.3% of the total number are practising in urban areas (Table 3). Even though Romanian legislation states a maximum of 2200 people per GP as the standard in primary healthcare, with an optimal number of 1800 people per GP in urban areas [90], these levels are rarely respected.

The shortage of specialists covers a wide range of medical specialities, with over 8600 vacancies for physicians reported in public hospitals as of early 2024 [91]. According to the NIS database, there are 2638 cardiologists nationwide, meaning 14 cardiologists per 100,000 inhabitants, significantly lower than the EU average (Table 3).

The shortage of medical personnel, corroborated by their geographic maldistribution, leaves many communities severely underserved. The emigration of medical professionals has also contributed to this crisis. Since Romania’s EU accession in 2007, many physicians have relocated to other European countries in search of better opportunities [61]. Despite recent increases in medical graduates and initiatives aimed at raising salaries and ameliorating working conditions, the healthcare system continues to struggle with a lack of physicians, particularly specialists.

### 3.2. Identifying Cardiovascular Deserts

**Figure 1:**
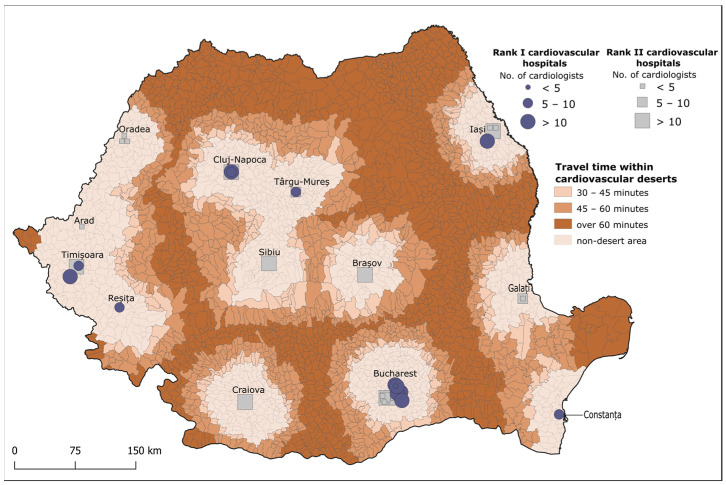
Cardiovascular deserts in Romania.

The add-ins ‘travel time’ and ‘distance’ of the API were used for measurements between all centroids of LAU2s and the closest identified hospital units. The criteria used in delineating cardiovascular deserts consisted of identifying LAU2s located farther than 60 km from a CV hospital that can provide complex care and from which the population had to travel for longer than 30 min to reach the hospital unit. Nationally, the CV deserts identified include 2223 LAU2s translating into 70% of the total LAU2s. The identified cardiovascular deserts contain 12,106,291 inhabitants, representing 64% of the national population (Figure 1 and Table 4).

The most extensive CV desert areas are located in the northeast part of the country and contain 16.3% of the total Romanian population. Also, sizeable cardiovascular desert areas are located in southern Romania. These concentrate more than 2 million inhabitants. Less extensive cardiovascular desert areas are found in the central and southeastern parts of the country and incorporate 5.9% and 7.2% of the total population, respectively (Table 4).

### 3.3. Hierarchisation of Cardiovascular Deserts

The classification of identified CV deserts was conducted by evaluating both population vulnerability to CVDs and their need for care, alongside an assessment of the existing healthcare infrastructure through multiple indicators. This initial classification served as a foundation for establishing a hierarchy that reflects the degree of cardiovascular desertification.

**Figure 2:**
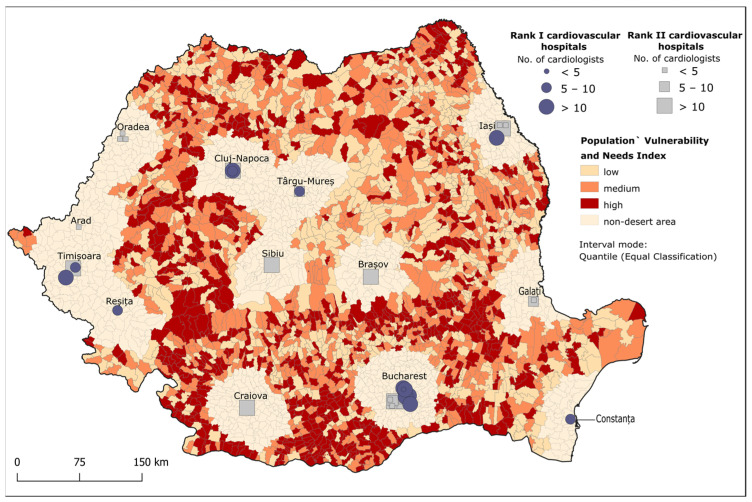
Classification of the population vulnerability to CVDs and potential needs for care.

Population vulnerability to CVDs and needs for CV care were assessed through *IV_n_*, considering the mortality rate due to CVDs, the percentage of the population over 65 years and the population density. High *IV_n_* values mean high mortality rates due to CVDs (over 2085 per 100,000 inhabitants). high percentages of the population over 65 years old (31–48%) and high density (2580–5283 inhabitants/km^2^) (Figure 2). High values (0.62–1.48) of the *IV_n_* were observed in peripheral areas, particularly in the country’s west, southwest and south. In contrast, low *IV_n_* values (0–0.48) comprise CV desert areas in the central part of the country (Figure 2 and Table 5).

**Figure 3:**
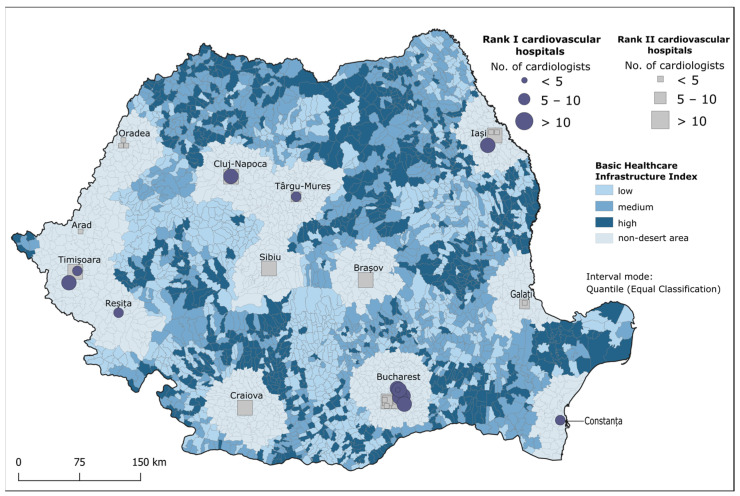
Classification of the supply of basic healthcare infrastructure.

The *IB_hi_* reflects the provision of basic healthcare infrastructure in all the LAU2 identified as cardiovascular deserts. Low *IB_hi_* values (0–0.14) illustrate a lack of healthcare infrastructure, such as GP offices, pharmacies and hospitals, which are predominant in areas from the western, southwestern and southern parts of the country. High *IB_hi_* values (0.44–5.1) indicate a higher number of available healthcare infrastructure and can be found in the southeast and northwest (Figure 3 and Table 6).

**Figure 4:**
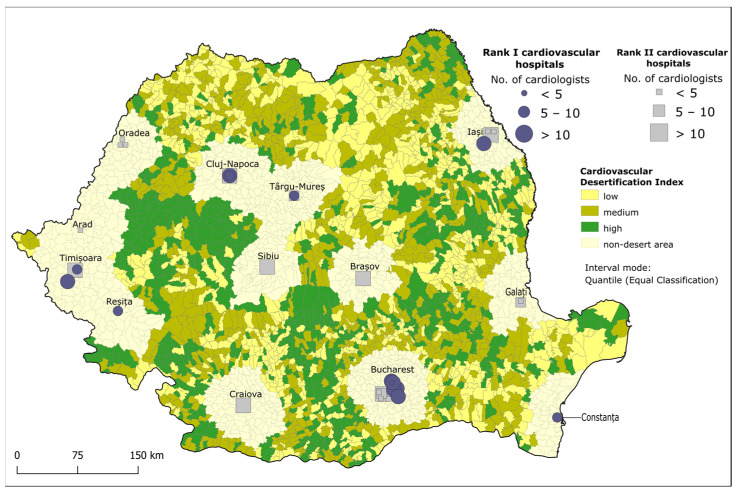
The degree of cardiovascular desertification in Romania.

The degree of cardiovascular desertification was evaluated through *ID_cv_*. High values (0.79–1) stand for the worst situation where a lack of healthcare infrastructure is combined with high population vulnerability to CVDs, while values close to 0 correspond to an improved situation where basic healthcare infrastructure is available and the vulnerability of the population to CVDs is lower. The south and northeast parts comprise CV desert areas with the highest number of inhabitants living in zones with a high degree of desertification, especially in rural and mountainous settings (Figure 4 and Table 7).

As opposed to this distribution, the northeast region comprises areas with the largest population affected by low degrees of cardiovascular desertification. In other parts of the country, the population lives in CV desert areas that cover all three degrees of desertification (Table 7).

## 4. Discussion

The Romanian healthcare system faces numerous and persistent problems. While repeated reforms have tried to emphasise the importance of primary care and enhance its role in healthcare provision, many times they have ended up chronically underfunding the system. This resulted in the impossibility of creating a solid network of primary care units and has kept the system hospital-centred. Additionally, small and predominantly rural hospitals could not obtain quality equipment or specialised healthcare staff to treat more complex issues successfully. As such, the populations living in those areas are generally redirected to larger cities with better-ranked hospitals, leading to increased pressure on their services [63]. The distribution of healthcare facilities is a prerequisite to access. Access to CV care is a complex and multifactorial issue that involves the availability of healthcare providers. Studies have shown that access to CV care remains a significant challenge in Romania [2,9,60,62]. As such, identifying shortages of CV care, that is, cardiovascular deserts, is a necessary step in reducing disparities and addressing social determinants of health. The analysis of cardiovascular deserts has implied a multidimensional perspective (geographic, population health status and facility-based infrastructure).

This research used both distance and travel to delineate cardiovascular deserts at the national level. These criteria, mirroring other medical desert studies [2,13,14], were a distance of more than 60 km to reach a hospital (in this case, a CV hospital able to provide complex care) and a travel time longer than 30 min. We also chose to create three travel time slots, which emphasised that 7,045,796 inhabitants must travel more than 60 min to receive complex CV care. It is important to note that the precariousness of these cardiovascular desert areas is primarily marked by Romania’s geographic features, as they span mostly mountainous and wetland areas, and secondarily by the poor national road infrastructure that does not efficiently cover the entire territory of the country. Furthermore, CV hospitals with specialised equipment and qualified cardiologists are unequally distributed across the country, which increases the desertification degrees of the CV desert areas identified.

Within the identified cardiovascular deserts, we calculated three distinct degrees of desertification to emphasise areas that require targeted intervention, such as strategic allocation of healthcare resources and tailored programs to mitigate CVD risk. In order to reach this hierarchy, we created two indexes. The first index, *IV_n_*, analyses the CV vulnerability of the population by incorporating indicators such as population density, mortality rate due to CVDs and percentages of the population over 65 years. Higher population density has been shown to coexist with better street connectivity and walkability and more options for increasing physical activity and improving nutrition [92]. Also, higher population density may facilitate social connections between individuals, which a US study found to strongly affect CV health [93]. Mortality due to CVDs has been correlated to population ageing [94]. This age segment is more prone to suffer from CVD complications and usually has limited financial resources, which, in addition to their mobility issues, means that travelling long distances or for a long time to receive specialised care will surely be a burden [26]. Lower *IV_n_* values designate cardiovascular deserts containing small shares of the population vulnerable to or in need of CV care. These areas are usually located in the proximity of a city, which translates into better access to primary and hospital healthcare services (Figure 2).

Conversely, high *IV_n_* values reflect cardiovascular deserts with low population density and higher mortality rates due to CVDs. High *IV_n_* values can be observed in the southwest of the country, where presently, the highest mortality values due to CVDs are registered. This part of the country contains only 11 municipalities with a low-rank hospital (III–V); as such, residents in need of CV care have to travel to neighbouring regions for specialised treatment, as shown in a 2016 report that found that 24% of residents received medical care elsewhere [95].

In studying cardiovascular deserts, it is important to observe not only the location of the most vulnerable population but also the supply of the necessary healthcare infrastructure. Consequently, we constructed the *IB_hi_* as an index that shows provider-to-population ratios in terms of GPs, GP offices and pharmacies, as well as the presence of a III, IV or V-ranked hospital.

The *IB_h_*_i_ reveals that peripheral regions are disproportionately affected by limited basic healthcare infrastructure. The underserving of peripheral areas has resurfaced with the COVID-19 pandemic, which showed the ease with which small hospitals can be overwhelmed [22]. In the southeast part of the country, the deltaic area makes transportation available only by boats, while in the southwest and west, mountains create difficulties in accessing the health infrastructure, thus reflecting low values of the *IB_h_*_i_ index (Figure 3). These conditions often force residents to travel longer distances for medical services, creating longer travel times and costs that add to the country’s infrastructural burden. Patients with CVD often rely on a regimen of medications, including antihypertensives, statins and anticoagulants, to manage their conditions effectively. A lack of pharmacies, for example, also emphasised by low values of *IB_hi_*, leads to treatment interruptions and increases the risk of CV events, such as heart attacks and strokes [96]. Low and medium values of *IB_hi_* mean that the respective cardiovascular deserts lack one or more of the healthcare infrastructure units included in this index. For example, the northeast part of Romania faces low values of *IB_hi_*, which, combined with its lower income levels and higher prevalence of poverty, makes it difficult for the population to travel long distances in order to access essential services such as healthcare [97]. An average of 24.6 GP consultations per day were recorded in 2011 for this part of the country [98].

The *ID_cv_* hierarchises the degree of cardiovascular desertification within the identified CV deserts and was constructed based on the relationship between *IV_n_* and *IB_hi_*. The main advantage of this index is that it is able to highlight areas where the population is highly vulnerable to CVDs and in need of specialised care, but the supply of basic healthcare infrastructure is unable to match this demand.

The increasing emigration of doctors and nurses, peaking in 2010, has jeopardised the proper running of many facilities, especially small municipal hospitals, with rural areas eventually facing the lowest coverage with medical staff [99]. The cardiovascular desertification of the Romanian territory is not only a result of the emigration of doctors but also of their ageing. In 2012, the average age of the GPs was 49.5 years [98], with 48% of them aged 60 and over, which means that in the next five years, Romania will have a bigger problem than the one presented in this study. Large parts of the country with high *ID_cv_*, mainly rural areas, are devoid of hospitals, highlighting a mismatch between population vulnerability to CVDs and existing basic healthcare infrastructure. This is the case in the country’s west, southwest and south parts, where the *IV_n_* has high values (0.62–1.48) while the *IB_hi_* has low values (0–0.14), resulting in a high degree of CV desertification (*ID_cv_*). Areas of low degrees of CV desertification usually include medium-sized cities and one or more low-ranked hospitals. The areas with high degrees of CV desertification tend to be farther from large urban centres, while low values characterise areas situated closer to cities with high-rank hospitals, such as those near Craiova, Oradea and Bucharest. This distribution of *ID_cv_* suggests that proximity to urban healthcare hubs significantly mitigates desertification (Figure 4).

CVDs require ongoing management, including lifestyle counselling, medication adjustments and routine follow-ups. An ageing population combined with a lack of adequate primary care provided by GPs can lead to more severe health issues and hospitalisation. Patients with unmanaged CVDs are more likely to experience acute events, such as heart attacks or strokes, which could have been prevented with regular GP visits and monitoring [100]. This can lead to poorly managed conditions and an increased risk of complications [101].

Numerous studies have concerned themselves with strategies to make areas affected by medical desertification more attractive for the health workforce [102,103,104,105]. In Romania, despite salary increases of up to 25% for GPs who choose to work in rural areas and 200% for those who select the Danube Delta, the government has not adequately addressed the retention of GPs in these areas. Hiring freezes and bureaucratic hurdles further complicate efforts to fill vacant positions [106]. Financial incentives alone are unlikely to attract a healthcare workforce to underserved areas, suggesting a combination of measures might be more successful [107]. For example, Günther et al. (2010) propose the availability of childcare facilities and fewer on-call duties [108]. Hancock et al. (2009) note that personal history with a place might play an important role; as such, organising internships in rural practices will help students understand the area’s specificity and develop empathy for the local community’s patients [103]. This can also be achieved through curriculum changes that account for rural practices while simplifying bureaucratic or restrictive measures. Another proposal refers to the provision, free or subsidised, of facilities, equipment or real estate needed for GPs to establish their practice [9].

Against the backdrop of the imposed isolation regulations created by the COVID-19 pandemic and technological progress, new methods of accessing healthcare have gained traction. Mobile videoconferencing systems have been shown many times to be cost-effective [109,110], while telemedicine consultation was found to improve patient care [111]. In Spain, pilot programmes implemented in rural aged populations have expanded due to their positive outcomes [112]. Using telemedicine or telehealth led to a drop in hospital visits in Texas [113]. While Romania’s legislation and healthcare systems are slow to embrace changes, sustained steps are being taken in this direction. Emergency Ordinance No. 18 [114] proposed the establishment of community healthcare services to compensate for the lack of health services in rural, isolated areas. However, by the following year, there were only 45 community healthcare centres in which more than 1500 community health nurses and 470 health mediators were working. Emergency Ordinance No. 196 (2020) promotes the use of telemedicine [115]. This transition is feasible as 80.8% of the population had access to the internet in 2021. In the same year, smartphone ownership increased to 67.2% and digital literacy reached 31% [116]. Another helpful step is the Law on Mobile Health Care (2022), which facilitates access to essential health services for people in rural areas with a shortage of doctors [117].

### Study Limit

This study has some limitations, the most important being the absence of a uniform healthcare database and the fragmentation of available data. Quantitative data on health services in Romania have to be gathered from various sources, which means this research faced numerous challenges. The validity of this research depends on the quality and comparability of the data acquired. The indexes will be iterative as data are updated and access to key national datasets improves. LAU2 level units are the smallest level at which one can obtain comparable data in Romania, which is the reason why this study used the centroid approach at the LAU2 level to measure distance and travel time to the nearest CV hospital that can provide complex care. While this approach has been used in numerous studies, the authors are aware that the best methodology will be using the household address of each individual in the population. Unfortunately, this is impossible to do at the national level in Romania. In the construction of the *IB_hi_* index, the authors considered the provider-to-population ratio using the entire population of an LAU2. However, there might be situations where proximity can also translate into people from one LAU2 going to a GP from a different one. Similarly, LAU2 without GPs or pharmacies will refer to healthcare units from a different LAU2. Also, while distance, travel time, existing healthcare infrastructure and population characteristics have been included in this study, cardiovascular deserts can also be influenced by organisational aspects such as GP opening hours as well as financial aspects (costs incurred for travelling or informal payments).

## 5. Conclusions

There is a scarcity of medical desert studies in Eastern Europe, certainly at the Romanian national level. Given that the main causes of death and morbidity in Romania are CVDs, a study on CV deserts is not only important but vital. As the first study on CV deserts in Romania, this research set out to identify the most vulnerable areas of the country considering multiple factors and indicators. Its premise was much more than to reiterate the existence of an urban–rural divide but rather to emphasise that a considerable population of the country lives in areas of low, medium or high desertification degrees. This study reveals that over 64% of the Romanian population face having to travel more than 30 min and live over 60 km away from a CV hospital able to provide complex CV care. Apart from proposing a methodology for identifying such areas, this study creates a hierarchy of cardiovascular desertification, which uses population vulnerability to CVDs and their need for CV care, as well as the supply of basic healthcare infrastructure. The degrees of cardiovascular desertification identified in this study, and especially the high values of *ID_cv_*_,_ allow for pinpointing areas where the population is highly vulnerable to CVDs but lacks the basic healthcare infrastructure to receive care.

The findings of this study underscore the areas in need of urgent targeted interventions to enhance healthcare access and reduce the burden of CVDs in Romania. Decision-makers can use our results to ensure equitable access to healthcare, addressing the disparities highlighted by the high values of the *ID_cv_*. Our study showed that these areas are primarily remote rural or mountainous parts in the west, southwest, southeast and northeast of Romania. The approach of this study is pertinent because it is the first study that incorporates travel time and distance to the nearest CV hospital with the vulnerability to particular diseases and analysis of healthcare infrastructure in specific areas, as well as the characteristics of the population living in those areas.

Future studies could explore the socio-economic factors influencing healthcare access in cardiovascular deserts, mainly how income and mobility issues affect patients’ ability to seek care. Implementing telemedicine and mobile healthcare services, as well as employing more community healthcare workers, could significantly improve access to specialised care in these underserved areas.

## Figures and Tables

**Table 1:** Correlation matrix.

	% pop. over 65 Years	Mortality Rate due to CVDs	Population Density
% pop. over 65 years	1		
mortality rate due to CVDs	0.547916137	1	
population density	−0.091009078	−0.185150548	1

**Table 2:** Healthcare infrastructure in Romania (2023).

Healthcare Units	Total	Urban Areas	Rural Areas
Hospitals	546	493	53
GP offices	10,266	6182	4084
Pharmacies	8247	5408	2839
Stomatology offices	16,946	14,535	2411
Total	36,005	26,618	9387

Source: NIS.

**Table 3:** Healthcare workforce in Romania (2023).

Medical Personnel	Total No.	Urban Areas (No.)	Rural Areas (No.)	Romania Inhabitants/ Medical Personnel (Average)	EU Inhabitants/Medical Personnel (Average)
Physicians	72,740	67,330	5410	258	225
out of which GP	12,471	8516	3955	1254	1125
Cardiologists	2638	-	-	-	-
Pharmacists	21,736	18,377	3359	1064	1095
Physicians	72,740	67,330	5410	258	225

Source: NIS and Eurostat.

**Table 4:** Population living in cardiovascular deserts in Romania.

	Population Living in CV Deserts	Population Living in CV Deserts as a % of the Total Population	No. of LAU2 Included in the CV Deserts
centre	1,118,877	5.9	212
north-east	3,091,196	16.3	434
north-west	1,521,945	8.0	300
south	2,670,006	14.1	480
south-east	1,364,702	7.2	272
west	2,328,038	12.3	525

**Table 5:** Population vulnerability to CVDs and potential needs for care based on *IV_n_* values.

	CV Deserts with High *IV_n_* Values		CV Deserts with Medium *IV_n_* Values		CV Deserts with Low *IV_n_* Values	
Location	No. of Inhabitants	% of the Total Population	No. of Inhabitants	% of the Total Population	No. of Inhabitants	% of the Total Population
centre	210,946	1.1	363,258	1.9	544,673	2.9
north-east	1,172,383	6.2	1,001,268	5.3	917,545	4.8
north-west	463,997	2.4	607,696	3.2	443,881	2.3
south	2,072,939	10.9	1,130,785	6	464,287	2.4
south-east	595,702	3.1	496,380	2.6	272,620	1.4
west	256,443	1.4	287,397	1.5	161,265	0.9

**Table 6:** Population in the identified CV deserts dependent on the supply of basic healthcare infrastructure.

	CV Deserts with High *IB_hi_* Values		CV Deserts with Medium *IB_hi_* Values		CV Deserts with Low *IB_hi_* Values	
Location	No. of Inhabitants	% of the Total Population	No. of Inhabitants	% of the Total Population	No. of Inhabitants	% of the Total Population
centre	564,997	3	206,700	1.9	358,647	1.9
north-east	1,688,572	8.9	760,949	5.3	641,675	3.4
north-west	800,891	4.2	428,208	3.2	295,316	1.6
south	2,207,057	11.6	1,386,937	6	1,037,020	5.5
south-east	680,545	3.6	432,916	2.6	251,241	1.3
west	358,608	1.9	158,705	1.5	187,792	1

**Table 7:** Population living in cardiovascular deserts based on degrees of desertification.

	High Degree of Desertification		Medium Degree of Desertification		Low Degree of Desertification	
Location	No. of Inhabitants	% of the Total Population	No. of Inhabitants	% of the Total Population	No. of Inhabitants	% of the Total Population
centre	300,663	1.6	260,365	1.4	557,849	2.9
north-east	575,394	3.0	788,176	4.2	1,727,626	9.1
north-west	243,366	1.3	502,418	2.6	7,761,661	40.9
south	634,863	3.3	677,556	3.6	1,357,587	7.2
south-east	304,150	1.6	334,459	1.8	721,254	3.8
west	602,213	3.2	610,526	3.2	115,259	0.6

## Data Availability

The data presented in this study are available upon request from the corresponding authors.
